# Significant Contribution of Mouse Mast Cell Protease 4 in Early Phases of Experimental Autoimmune Encephalomyelitis

**DOI:** 10.1155/2016/9797021

**Published:** 2016-08-17

**Authors:** Louisane Desbiens, Catherine Lapointe, Marjan Gharagozloo, Shaimaa Mahmoud, Gunnar Pejler, Denis Gris, Pedro D'Orléans-Juste

**Affiliations:** ^1^Department of Pharmacology, Medical School, Université de Sherbrooke, 3001 12th Avenue North, Sherbrooke, QC, Canada J1H 5N4; ^2^Department of Pediatrics, Program of Immunology and Allergology, Medical School, Université de Sherbrooke, 3001 12th Avenue North, Sherbrooke, QC, Canada J1H 5N4; ^3^Department of Medical Biochemistry and Microbiology, Uppsala University BMC, Box 582, 75123 Uppsala, Sweden; ^4^Department of Anatomy, Physiology and Biochemistry, Swedish University of Agricultural Sciences, P.O. Box 7070, 75007 Uppsala, Sweden

## Abstract

Experimental autoimmune encephalomyelitis (EAE) is a mouse model that reproduces cardinal signs of clinical, histopathological, and immunological features found in Multiple Sclerosis (MS). Mast cells are suggested to be involved in the main inflammatory phases occurring during EAE development, possibly by secreting several autacoids and proteases. Among the latter, the chymase mouse mast cell protease 4 (mMCP-4) can contribute to the inflammatory response by producing endothelin-1 (ET-1). The aim of this study was to determine the impact of mMCP-4 on acute inflammatory stages in EAE. C57BL/6 wild type (WT) or mMCP-4 knockout (KO) mice were immunized with MOG_35–55_ plus complete Freund's adjuvant followed by pertussis toxin. Immunized WT mice presented an initial acute phase characterized by progressive increases in clinical score, which were significantly reduced in mMCP-4 KO mice. In addition, higher levels of spinal myelin were found in mMCP-4 KO as compared with WT mice. Finally, whereas EAE triggered significant increases in brain levels of mMCP-4 mRNA and immunoreactive ET-1 in WT mice, the latter peptide was reduced to basal levels in mMCP-4 KO congeners. Together, the present study supports a role for mMCP-4 in the early inflammatory phases of the disease in a mouse model of MS.

## 1. Introduction

Mast cells are integrally involved in cellular based immune responses to pathogens as well as inflammatory reactions prompted by pathogens or toxins [1, 2] and have been suggested for several years to play a part in the acute phase of Multiple Sclerosis (MS) [3–8]. Albeit the mechanisms by which mast cells influence MS are yet to be fully understood, trypsin-like proteases released from degranulating mast cells have been shown to trigger demyelination in a mouse model for MS-experimental autoimmune encephalomyelitis (EAE) [9]. Data from Secor et al. (2000), furthermore, support that mast cells are involved in the pathogenesis of EAE [3]. The same group has more recently suggested a significant role for mast cells in activation of inflammasomes localized within meninges [10]. In contrast, other groups have challenged the contribution of mast cells in EAE [11–13]. In particular, Feyerabend and colleagues show that the complete ablation of MCs in a “kit independent” MC-deficient strain does not affect EAE development [12]. Albeit targeting mast cells as a viable approach to alleviate the disease remains debatable, the contribution of mast cell-derived proteases in EAE is still largely uninvestigated. One of the many mast cell-expressed proteases that potentially may account for the contribution of mast cells to MS/EAE is mouse mast cell protease 4 (mMCP-4), a *β*-chymase predicted to be the murine functional counterpart to the single human chymase (CMA1) based on deduced amino acid sequence, tissue localization, and serglycin storage dependence [14, 15]. In support for a role of this chymase in mast cell-dependent inflammatory conditions, mMCP-4 plays a protective role in a mouse model of mechanically induced cerebral trauma [16], yet it is detrimental in lung inflammation and immune complex-induced glomerulonephritis [17, 18]. In further support for a role of mMCP-4 in regulating inflammatory mediators, our group has reported that mMCP-4 generates endothelin-1 (ET-1) from its precursor big-ET-1 [19, 20] and that mMCP-4 knockout (KO) mice display a 40% reduction in pulmonary ET-1 levels when compared to wild type (WT) congeners [19].

The role of ET-1 as a marker in the etiology of MS has only been explored in a limited fashion. Haufschild et al. (2001) reported a significant increase in ET-1 plasma levels in untreated MS patients [21], an observation confirmed by Pache and colleagues (2003) [22]. In treated MS patients though, such increases were not found [23]. However, in further support for a role of ET-1 in MS, ET-1 was shown to be overexpressed in a murine model of EAE [24].

Based on the above-suggested links between ET-1 and MS and between chymase and ET-1 generation, respectively, we here asked whether chymase might have a role in EAE and whether that enzyme in this experimental setting has a regulatory effect on ET-1 production. Indeed, the findings presented here suggest that mMCP-4 has a significant detrimental impact on the course of EAE and plays role in the generation of ET-1 in this mouse model for MS. The impact of mMCP-4 in EAE introduces a potential role for mast cell chymase in MS and thereby identifies the inhibitors of this particular enzyme as potential targets for therapy of MS.

## 2. Material and Methods

### 2.1. Mice

C57BL/6 mice (i.e., wild type (WT)) were purchased from Charles River Canada (Montréal, QC, Canada) and housed in our local facility. mMCP-4 KO mice genitors were provided by Dr. Gunnar Pejler (Uppsala University, Sweden) and were bred in our facility. The mMCP-4 KO mice have been backcrossed for over 10 generations with C57BL/6 congeners and are therefore highly congenial with the later strain [25]. Finally, the genotype of mMCP-4 KO mice used in the present study was confirmed by polymerase chain reaction (PCR) (as shown in Supplementary Figure  1 in Supplementary Material available online at http://dx.doi.org/10.1155/2016/9797021) via the use of primers described in Supplemental Table  1. We had previously reported, in mMCP-4 KO mice* in vivo* as well as in tissues or mastocytes derived from this mouse strain, the complete loss of chymase-dependent hydrolytic activity [19, 20].

All animals were kept at constant room temperature (23°C) and humidity (78%) under a controlled 10–14 h light/dark cycle. Mice had free access to standard chow and tap water* ad libitum*. Animal care and experimentation were approved by Ethics Committee on Animal Research of the Université de Sherbrooke in accordance with the guidelines of the Canadian Council on Animal Care.

### 2.2. Experimental Autoimmune Encephalomyelitis (EAE)

Induction of EAE was performed according to the protocol of Miller and Karpus (2007) [26]. In brief, a 1 : 1 emulsion mixture of myelin oligodendrocyte glycoprotein (MOG_35–55_) (Genemed Synthesis Inc., San Antonio, TX, USA) and complete Freund's Adjuvant (CFA) (Sigma-Aldrich, St. Louis, MO, USA) supplemented with 100 *μ*g of heat-killed* Mycobacterium tuberculosis* H37RA (Difco Laboratories, Detroit, MI, USA) was prepared. Female mice, at 8–10 weeks old, were subcutaneously injected in two sites (100 *μ*L by site) adjacent to the tail with the emulsion. Pertussis toxin (200 ng) (List Biological Laboratories Inc., Campbell, CA, USA) was administered intraperitoneally on the same day of immunization. Mice were scored daily with the following scale to assess clinical scores: 0, no sign of clinical disease; 0.5, partial tail paralysis (loss of tip tail tonus); 1, tail flaccidity or hind limb weakness; 2, limp tail and weakness in limb; 3, partial hind limb paralysis; 4, total hind limb paralysis; and 5, moribund state or death.

### 2.3. Histopathology

The immunized mice were anesthetized by intraperitoneal injection of 2,2,2- tribromoethanol (Avertin) (approximately 240 mg/kg) (Sigma-Aldrich, St. Louis, MO, USA), prepared in tert-amyl alcohol and diluted in 0,9% saline solution. The mice were then perfused with ice-cold PBS buffer (Wisent, St. Bruno, QC, Canada), and the spleen, cervical, and thoracic spinal cords and left and right brain hemispheres were collected and stored at −80°C immediately, whereas lumbar spinal cords were placed in 10% buffered formalin phosphate (Fisher Scientific, Waltham, MA, USA) before being embedded in paraffin and cut into 5 *μ*m sections.

### 2.4. Histology Stains

Slides were deparaffinised in xylene (Electron Microscopy Sciences, Hatfield, PA, USA) and hydrated in 100, 95, and 70% ethanol gradient followed by water.

#### 2.4.1. Toluidine Blue (Mast Cells)

Slides were incubated 2-3 minutes at room temperature in toluidine blue (Sigma-Aldrich, St. Louis, MO, USA) working solution prepared by dilution of stock solution (1% in ethanol 70%) in sodium chloride 1% pH 2–2.5. The slides were rinsed in water and quickly dehydrated in 95–100% ethanol before being rinsed with xylene and coverslipped with Permount (Fisher Scientific, Ottawa, ON, Canada).

#### 2.4.2. Luxol Fast Blue (Myelin)

Slides were incubated overnight at 56°C in 0.1% luxol fast blue solution (Electron Microscopy Sciences, Hatfield, PA, USA) in 95% alcohol w/acetic acid. The slides were then rinsed in distilled water before being differentiated in lithium carbonate 0.05% and in 70% ethanol for approximately 30 seconds each, since the grey matter is clear and white matter is sharply defined. The slides were rinsed in water and dehydrated in 100% ethanol before being cleared in xylene and coverslipped with Permount.

All histological slides were scanned with a NanoZoomer 2.0-RS Digital slide scanner (Hamamatsu Photonics, Shizuoka, Japan) before being treated with the NDP.view2 Viewing software and paint.net software and staining density was quantified by ImageJ 1,49V (Wayne Rasband, NIH, USA).

#### 2.4.3. Immunofluorescence for Iba1 (Microglia) and GFAP (Astrocytes)

Prior to measuring immunofluorescence, with a Sequenze Slide Rack and Coverplate System (Ted Pell Inc, Redding, CA, USA), an antigen unmasking was performed by a 10 minutes' incubation in 10 mM sodium citrate buffer pH 6.0 (Sigma-Aldrich, St. Louis, MO, USA) at a subboiling temperature. The slides were then washed in 0.1% Triton X-100 in PBS solution and blocked in 5% fetal bovine serum (FBS) supplemented with 0.1% Triton X-100 in PBS for one hour before being incubated overnight at 4°C with primary antibody (1 : 1000), against the rabbit anti-glial fibrillary acidic protein (GFAP) (Cedarlane, Burlington, ON, Canada) or against the rabbit anti-ionized calcium binding adaptor molecule 1 (Iba1) (Wako, Osaka, Japan). A 2 hours' incubation at room temperature with the secondary antibody (1 : 2000), Alexa Fluor 488 AffiniPure Goat Anti-Rabbit IgG (H + L) (Jackson ImmunoResearch Laboratories Inc., West Grove, PA, USA), was then performed. The slides were mounted with DAPI Fluoromount-G (SouthernBiotech, Birmingham, AL, USA) and photomicrograph pictures were taken with Retiga SRV Mono Cooled numerical camera attached to Zeiss Axioskop 2 Microscope. The pictures were stitched with Adobe Photoshop CS3, and stain density was quantified with Image-Pro Plus 5.1 (Media Cybernetics Inc., Rockville, MD, USA).

### 2.5. Measurement of Endogenous Brain ET-1 Levels

The left parts of brain from healthy or 1, 2, or 3 weeks post-EAE-induced mice were homogenized in a chloroform : methanol (1 : 4) solution and then purified on a DSC-18 solid phase extraction column (Supelco, Bellefonte, PA, USA) and eluted in acetonitrile : water : trifluoroacetic acid (ACN 60% : H_2_O 40% : TFA 0,1%). The collected eluates were then Speed Vac-dried overnight before reconstitution in PBS supplemented with 1/32 mouse plasma and endogenous ET-1 was measured by Quantikine ELISA kit from R&D systems (R&D systems, Minneapolis, MN, USA) according to the manufacturer's instructions.

### 2.6. Measurement of Spinal Cord Endogenous Interferon-Gamma (IFN*γ*) Levels

Tissue lysates were prepared as described previously [27]. Briefly, frozen thoracic spinal cords were weighed and homogenized in 0.5 mL of ice-cold lysis buffer (Cell Signaling Technology, Beverly, MA, USA) supplemented with protease inhibitors (Roche Diagnosis, Mannheim, Germany) by rapid agitation for 2 minutes in the presence of 3 mm stainless beads. The tissue lysate was centrifuged for 20 minutes at 13,000 ×g at 4°C, and the supernatant was transferred to a new tube. The tissue levels of IFN*γ* were determined using murine ELISA development kits (PeproTech, Rocky Hill, NJ, USA), according to the manufacturer's instructions. The level of IFN*γ* was reported as pg/mg of tissue.

### 2.7. RNA Extraction and Quantitative RT-PCR

RNA from the right brain hemisphere derived from healthy or 1 or 2 weeks post-EAE-induced mice were extracted using RiboZol*™* reagent (Amresco Inc., Solon, OH, USA). Tissues were homogenized with a glass-Teflon homogenizer. Chloroform (200 *μ*L) (J.T. Baker, Central Valley, PA, USA) was added to each tube per 1 mL of RiboZol and incubated at room temperature for 3 minutes followed by centrifugation at 12,000 ×g for 15 minutes at 4°C. Nonopaque supernatants were collected and 500 *μ*L of isopropanol (Fisher Scientific, Ottawa, ON, Canada) was added for RNA precipitation and incubated 10 minutes at room temperature followed by centrifugation at 12,000 ×g for 10 minutes at 4°C. Pellets were washed with addition of 1 mL ethanol 75% followed by 7,500 ×g centrifugation at 4°C for 5 minutes before being redissolved in 50 *μ*L of DEPC water and an incubation at 55°C for 10 minutes. RNA concentration has been determined by absorbance at* A*_260_ and purity by ratio* A*_260_/*A*_280_. 1 *μ*g of RNA was then used for the remaining experiments. cDNA was synthesized using oligo(dT)_12–18_ primers (Invitrogen, Carlsbad, CA, USA), dNTPs mix 10 *μ*M each (Thermo Scientific, Waltham, MA, USA), in SuperScript III buffer with DTT, RNaseOUT, and SuperScript III (Invitrogen, Carlsbad, CA, USA). Quantitative PCR was performed for actin and mMCP-4 by monitoring in real time the fluorescence increase of the SYBR Green in the Perfecta SYBR Green SuperMix, low ROX (Quanta Biosciences, Gaithersburg, MD, USA) using the MX3000P Multiplex Quantitative PCR System (Agilent Technologies, Santa Clara, CA, USA). Primers (IDT, Coralville, IA, USA) were used at final concentration of 50 nM per primer and sequences were designed as follows:

 mMCP-4 F: 5′-CTCTCTCCAAGCTGTGACCGAC-3′,
 mMCP-4 R: 5′-CTATGAGCTCCAAGGGTGACA-3′,
 
*β*-actin F: 5′-GATCAAGATCATTGCTCCTCCTGAGC-3′,
 
*β*-actin R: 5′-GCAGCTCAGTAACAGTCCGCCTAG-3′.

mMCP-4 KO mice samples were tested as negative controls. As *β*-actin levels were stable between healthy and immunized mice, the latter mRNA was used as internal control for normalization and relative expression of chymase mMCP-4 was calculated using the 2^−ΔΔCt^ method.

### 2.8. Statistical Analysis

All data are presented as the mean ± SEM. All statistical analyses were conducted using GraphPad Prism 6 software (GraphPad Software, La Jolla, CA, USA). Statistical significance was reached when the “*p*” value was below 0.05 and determined using one-way ANOVA Kruskal-Wallis followed by Bonferroni for EAE clinical score or based on one-way or two-way ANOVA and multiple Student's* t*-test for all other analyses.

## 3. Results

### 3.1. Reduced Impact of EAE in mMCP-4 KO Mice When Compared to WT Congeners

**Figure 1:**
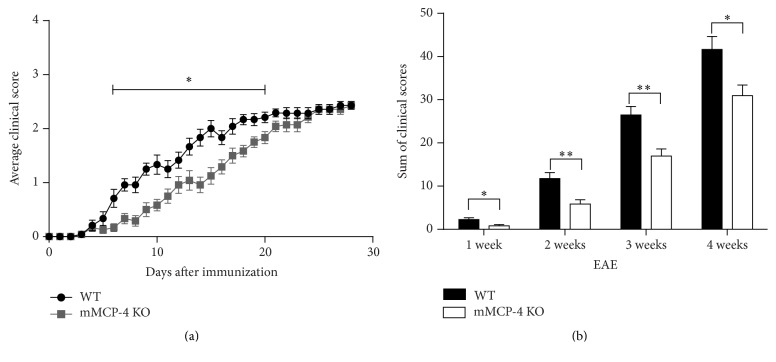
Delayed development of EAE symptoms without change in the severity of the disease in mMCP-4 KO mice. (a) Clinical score of WT (•) or mMCP-4 KO (▪) mice. (b) Sum of clinical score at different set points after immunization of WT (closed bars) and mMCP-4 KO (opened bars) mice. Each point or bar represents the mean ± SEM of 12 (weeks 1 to 3) and 7 mice (4th week). ^*∗*^ *p* < 0.05, ^*∗∗*^ *p* < 0.01.

An emulsified mixture of MOG_35–55_ in CFA was subcutaneously injected in WT or mMCP-4 KO mice. WT mice developed the disease approximately after 9 days whereas the mMCP-4 KO mice demonstrated a substantial delay in the appearance of clinical symptoms, being detectable starting from ~day 12 after injection (Figure 1(a)). Moreover, the development of severe disease (disease score 2.5) was markedly delayed in mMCP-4 KO mice up until the third week posttreatment as indicative of tail and back limb weaknesses. However, 4 weeks after EAE, WT and mMCP-4 KO mice exhibited similar disease scores with no evidence of total hind limb paralysis. A summation of the total clinical scores, on the other hand, supported a significant role of mMCP-4 as a pathogenic factor in EAE (Figure 1(b)), even up to the 4th week of observations (WT 41.64 ± 3.02; mMCP-4 KO 30.93 ± 2.48, *p* < 0.05, *n* = 7 mice).

**Figure 2:**
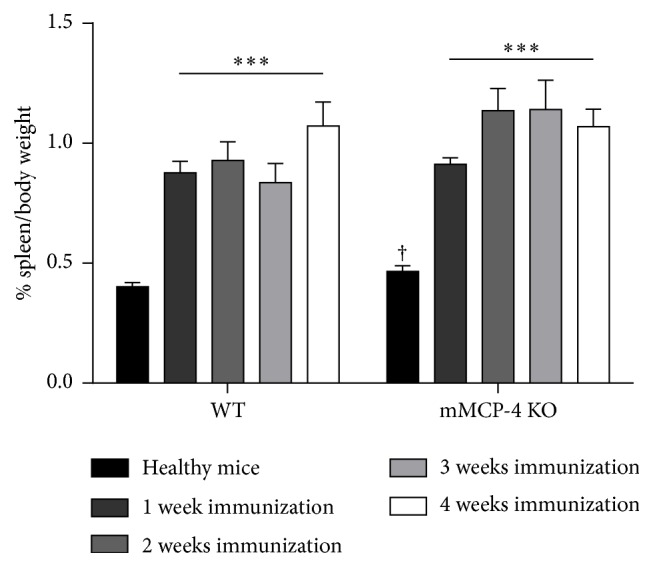
Induction with MOG/CFA induced an increase of spleen weight compared to healthy mice. Each bar represents the mean ± SEM of 6 to 15 mice. ^*∗∗∗*^ *p* < 0.001 versus healthy WT or healthy mMCP-4 KO mice or ^†^ *p* < 0.05 comparing WT versus KO mice at each time point studied.

To assess, on the other hand, whether mMCP-4 has an impact on the immune response, spleen weights of WT and mMCP-4 KO mice were monitored up to 4 weeks after immunization. However, as seen in Figure 2, spleen weights did not differ between the two genotypes except in healthy mice where it was significantly increased in mMCP-4 KO congeners (*p* < 0.05).

### 3.2. mMCP-4 KO Mice Demonstrate Lower Percentage of Reactive Gliosis after EAE

**Figure 3:**
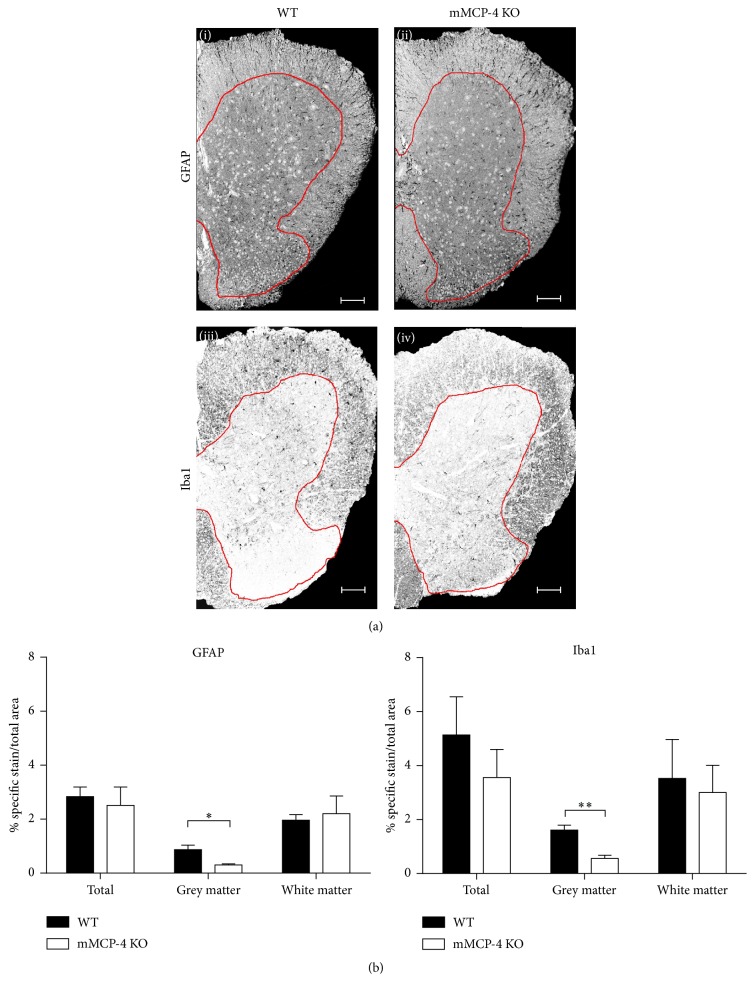
(a) Photomicrograph of the spinal cords stained with GFAP or Iba1. White and grey matters are delimited by the red line. Scale bar is 200 *μ*m. Upper panels: GFAP marking in (i) WT mice, 2 weeks after EAE, or (ii) mMCP-4 KO mice, 2 weeks after EAE. Lower panel: Iba1 staining in (iii) WT mice, 2 weeks after EAE, or (iv) mMCP-4 KO mice, 2 weeks after EAE. (b) Grey matter levels of microglia and astrocytes are decreased in mMCP-4 KO mice 2 weeks after EAE. Left panel, percentage of astrocytes in 2 weeks post-EAE-induced mice; right panel, percentage of microglia 2 weeks after EAE. ^*∗*^ *p* < 0.05 or ^*∗∗*^ *p* < 0.01 for EAE-induced WT versus EAE-induced mMCP-4 KO mice. Each bar represents the mean ± SEM in 4 mice, in duplicate.

In response to spinal cord insults, GFAP (an intermediate filament protein expressed by astrocytes and ependymal cells among others) is upregulated in the CNS [28]. As another sign of CNS damage, the reactive microglial response can be measured by the extent of upregulation of Iba1 [29]. To evaluate whether mMCP-4 can influence the levels of these markers of CNS damage, the spinal cords of 2 weeks after EAE immunized mice were extracted and stained for GFAP and Iba1 (Figure 3(a)). mMCP-4 KO mice and WT littermates showed no significant differences in percent level of astrogliosis (GFAP staining intensity) and microgliosis (Iba1 staining intensity) in the total area of the lumbar spinal cord or in the white matter (Figure 3(b)). In contrast, significant reductions in percentages of microgliosis and astrogliosis were found in the grey matter of mMCP-4 KO mice when compared to WT congeners, 2 weeks after EAE immunization (in WT and mMCP-4 KO mice, respectively.; astrogliosis in percentage: 0.87 ± 0.17 and 0.30 ± 0.04, *p* < 0.05; microgliosis in percentage: 1.61 ± 0.18 and 0.55 ± 0.12, *p* < 0.01) (Figure 3(b)).

**Figure 4:**
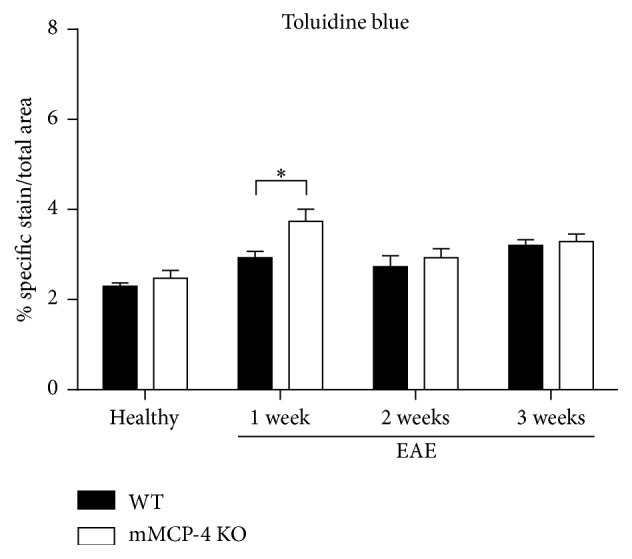
Mast cells density is increased in mMCP-4 KO mice on the first week after EAE only. Results are presented as the mean ± SEM of specific toluidine blue stained in white matter area. Each spinal cord was quantified in duplicate or triplicate for *n* = 4 mice. ^*∗*^ *p* < 0.05 versus WT mice EAE immunized.

To assess whether the absence of mMCP-4 has an influence on the number of mast cells, lumbar spinal cords from WT and mMCP-4 KO mice were stained with toluidine blue, both at baseline and after induction of EAE. As seen in Figure 4, the numbers of mast cells were similar in spinal cords from WT and mMCP-4 KO mice at the baseline state (healthy) and 2-3 weeks after induction of EAE. Notably though, there was a significant elevation of the numbers of mast cells 1 week after EAE (in WT and mMCP-4 KO mice, respectively; in percentage, healthy state: 2.30 ± 0.07 and 2.47 ± 0.17, 1 week after EAE: 2.93 ± 0.14 and 3.74 ± 0.27, *p* < 0.05, 2 weeks after EAE: 2.73 ± 0.25 and 2.93 ± 0.20, and 3 weeks after EAE: 3.20 ± 0.13 and 3.29 ± 0.17).

### 3.3. mMCP-4 KO Mice Show Higher Levels of Spinal Cord Myelin

**Figure 5:**
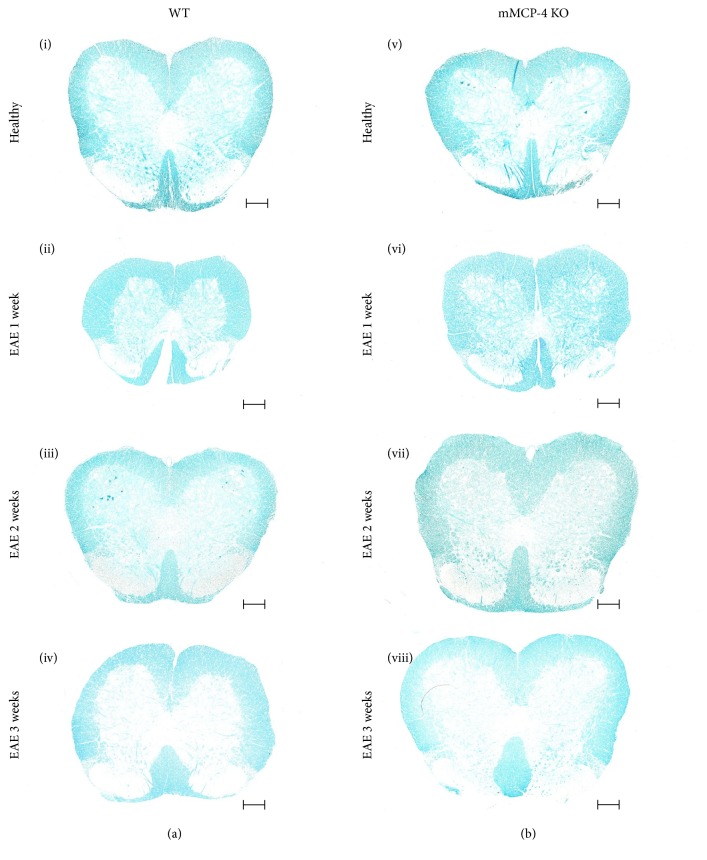
Photomicrograph of the spinal cords stained for assessment of myelin integrity with luxol fast blue. Scale bar is 200 *μ*m. (a) (i) WT mice, healthy, or (ii) 1 week after EAE, (iii) 2 weeks after EAE, and (iv) 3 weeks after EAE; (b) (v) mMCP-4 KO mice, healthy, or (vi) 1 week after EAE, (vii) 2 weeks after EAE, and (viii) 3 weeks after EAE.

**Figure 6:**
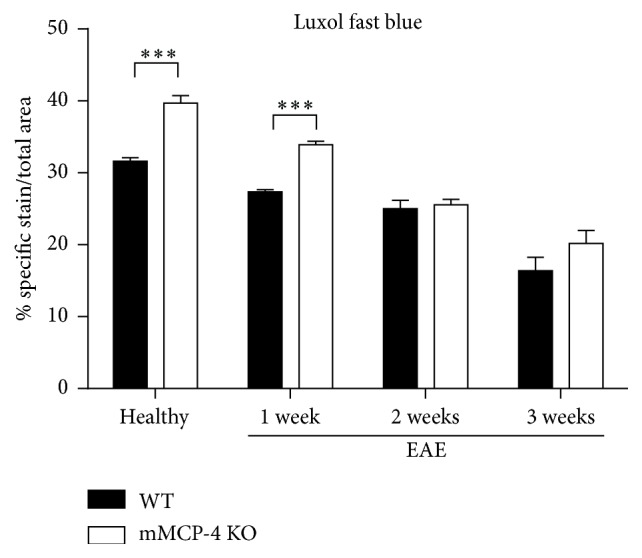
Myelin lost during the progression of EAE disease in healthy or 1, 2, or 3 weeks after EAE in WT mice (closed bars) and mMCP-4 KO mice (opened bars). Results are shown as the mean ± SEM of 4 mice quantified in duplicate or in triplicate. ^*∗∗∗*^ *p* < 0.01.

To assess whether chymase has an impact on the levels of intact myelin, lumbar spinal cords from WT and mMCP-4 KO mice were extracted and stained with luxol fast blue, a dye that stains myelin (Figure 5). Quantification of the luxol fast blue staining intensity revealed a significantly higher myelin content in mMCP-4 KO mice as compared with WT congeners, in both nonimmunized (healthy) mice and immunized mice 1 week after EAE induction (Figure 6) (in percentage, healthy WT mice: 31.62 ± 0.46 and healthy mMCP-4 KO mice: 39.70 ± 1.03, *p* < 0.001; WT 1 week after EAE: 27.37 ± 0.29 and mMCP-4 KO mice 1 week after EAE: 33.88 ± 0.50, *p* < 0.001). A subsequent decline in spinal cord myelin was seen in both WT and mMCP-4 KO mice 2 to 3 weeks after immunization as well as a loss of significant differences in myelin content between the two strains of mice (Figure 6).

### 3.4. EAE Increases Brain mMCP-4 mRNA and ET-1 Levels in WT Mice

**Figure 7:**
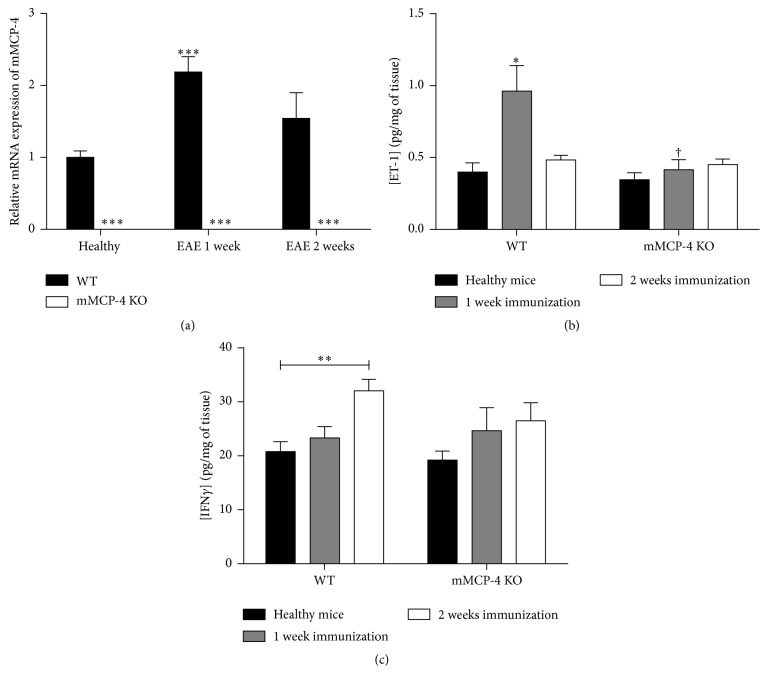
mMCP-4 brain mRNA expression is increased 1 week after EAE and is correlated with increase of ET-1 brain levels whereas INF*γ* is enhanced 2 weeks after EAE in spinal cord of WT but not mMCP-4 KO mice. (a) mRNA relative expression of mMCP-4 in healthy or 1 or 2 weeks after EAE quantified in WT (closed bars) and in mMCP-4 KO (opened bars) mice. (b) ET-1 levels of healthy (closed bars), 1 week (grey shaded bars), or 2 weeks after EAE (opened bars) in WT and mMCP-4 KO mice. (c) INF*γ* levels of healthy (closed bars), 1 week (grey shaded bars), or 2 weeks after EAE (opened bars) in WT and mMCP-4 KO mice. Each bar represents the mean ± SEM of 4 to 8 mice. ^*∗*^ *p* < 0.05; ^*∗∗*^ *p* < 0.01; ^*∗∗∗*^ *p* < 0.001 compared to WT mice or ^†^ *p* < 0.05 comparing WT versus KO mice at each time point studied.

To investigate if EAE is associated with an induction of the mMCP-4 gene, right brain homogenates derived from WT mice were analysed for mMCP-4 mRNA levels by RT-qPCR. As seen in Figure 7(a), a 2.2-fold increase in mMCP-4 mRNA expression (as compared with baseline levels in healthy mice) was found in WT mice one week after EAE immunization but not at later time points. Finally, EAE immunization promoted a twofold increase in mature ET-1 levels in left brain homogenates of WT but not mMCP-4 KO mice, one but not two weeks after EAE when compared to healthy WT mice (Figure 7(b)).

### 3.5. EAE Increases IFN*γ* Levels in Thoracic Spinal Cords

To investigate the immune response to EAE induction, we proceeded to ELISA quantification of IFN*γ* in thoracic spinal cord in healthy and 1 or 2 weeks after EAE. As shown in Figure 7(c), the levels of this cytokine are increased after EAE compared to healthy basal levels in both strains of mice, but significantly only in WT mice 2 weeks after EAE (quantities in pg/mg of tissue, healthy WT mice: 20.77 ± 1.84; WT 1 week after EAE: 23.30 ± 2.11; WT 2 weeks after EAE: 32.07 ± 2.11, *p* < 0.001 and healthy mMCP-4 KO mice: 19.23 ± 1.65; mMCP-4 KO mice 1 week after EAE: 24.66 ± 4.24; mMCP-4 KO mice 2 weeks after EAE: 26.47 ± 3.39).

## 4. Discussion

The main results of the present study are that mMCP-4 KO mice subjected to EAE show a reduced clinical score as well as brain levels of immunoreactive ET-1 when compared to WT congeners. In addition, the KO mice show reduction in percentages of microgliosis and astrogliosis as well as grey matter alterations.

These results therefore support our hypothesis that this particular chymase isoform plays a significant role in the early development of MS in the mouse EAE model. In our hands, the KO of mMCP-4 significantly reduced clinical scores up until 21 days after EAE induction.

It is noteworthy that mature mast cells can express numerous tryptases (having trypsin-like cleavage specificity) and chymases (having chymotrypsin-like cleave specificities) as well as other types of proteases [30]. Out of these, mMCP-4 was shown here to have a major impact on the course of EAE development. This suggests that mMCP-4 accounts to a major extent for the demyelination attributed to mast cells in EAE. This does not exclude, on the other hand, the involvement of other mast cell-derived mediators in this mouse model of MS. It is also of interest that EAE prompted a significant increase in ET-1 brain levels one week after EAE induction in WT but not in mMCP-4 KO mice. Notably, brain levels of this potent vasoactive peptide progressively returned to basal levels on the second and third weeks after EAE (results not shown for the 3rd week). These results suggest that ET-1 may be either an early marker or, alternatively, an early mediator of the inflammatory reaction occurring in the EAE model. Interestingly, Shin et al. (2001) reported that an ET_A_ antagonist, BQ-123, reduced the duration of paralysis, in EAE-induced rats [31]. The same authors also reported high levels of immunoreactive ET-1 in spinal cord-localized inflammatory and neuroglial cells [31]. These observations are of relevance considering that Hammond et al. (2014) have recently shown that ET-1 is a negative regulator of oligodendrocyte progenitor cell-dependent repair of demyelinated lesions [32]. In support of the latter observation, myelin contents were, in the present study, found to be higher in naive and even one week after EAE immunized mMCP-4 KO mice than in their WT congeners. Thus, interfering with the chymase-dependent production of ET-1 in MS may be a relevant therapeutic strategy in autoimmune diseases such as MS [33]. Noteworthy, chymase inhibitors have been shown to possess anti-inflammatory and antiproliferative properties in other settings [34–37].

Albeit still controversial, one should also note the beneficial effects of ET-1 antagonists on cerebral blood flow of MS patients [38]. Thus, ET-1 antagonists may have beneficiary effects not only by repressing cellular events in EAE but by improving overall brain blood circulation as well. Further studies currently ongoing in our laboratory should shed further light on the contribution of ET-1 via one ET_A_ and/or ET_B_ receptors and their associated mechanisms, in the etiology of EAE in the mouse model.

Albeit we show in the present study that mMCP-4 is detrimental in an experimental model of MS, the same concept cannot be extended to all types of experimental injuries of the CNS. For example, Hendrix and colleagues (2013) reported the protective role of mast cells (in part due to mMCP-4) in a mouse model of mechanically induced brain injury [16]. Noteworthy, in the same study the authors reversed the protective role of mMCP-4 with a general chymotrypsin inhibitor, chymostatin, which is nonspecific for chymase-dependent processes [16]. It has been elegantly reported by Kerschensteiner et al. (2004) that, in contrast to spinal cord injury models, EAE-induced lesions may facilitate additional levels of axonal reorganization based on the unlesioned fibers of the tract [39]. These observations support the concept that neuroinflammatory and surgical hemisections of the spinal cord, in the mouse model, alter in opposite fashion the “reserve capacity” of the CNS to regenerate [39]. Thus, we suggest that immune-related inflammatory reactions and surgical trauma in the CNS prompt opposite roles for mMCP-4.

Interestingly, monitoring of INF*γ* in our model suggests an enhanced inflammatory reaction in the spinal cord of WT but not mMCP-4 KO mice. We observed a significantly higher concentration of IFN*γ* in the spinal cords of WT compared to mMCP-4 KO mice 2 weeks after immunization. The decreased inflammatory response is correlated with the observation that mMCP-4 KO mice have decreased proliferation of microglia and astrocytes. The exact mechanism of such decreases is unknown. It can nonetheless be explained by decreased responses of CNS inflammatory cells in mMCP-4 KO mice which lead to the decreased concentration of proinflammatory molecules, decreased chemotactic gradient, decreased influx of Th1 cells, decreased IFN*γ*, and, as a result, decreased demyelination. On the other hand, preserved integrity of BBB may lead to reduced influx of Th1 cells from the periphery, a decreased activation of astrocytes and microglia, a decreased overall inflammation in the CNS, and a decreased demyelination. Based on the above observations, the present study reinforces the hypothesis suggested by Scandiuzzi et al. (2010), Reber et al. (2014), and Magnusson et al. (2009) [17, 18, 40] that mMCP-4 activates cells involved in tissue specific autoimmune reactions.

Overall we show in the present study that a single mouse chymase isoform, mMCP-4, contributes significantly to the etiology and early symptoms associated with a mouse model of MS, namely, EAE. If what is reported here in the mouse model can be extended to the clinical situation, it is suggested that targeting chymase rather than the overall mastocytic activity in that particular autoimmune disease will constitute an added value within the currently available therapeutic arsenal against this neurodegenerative disease.

## Supplementary Material

The mMCP-4 KO mice have been backcrossed for over 10 generations with C57BL/6 congeners and are highly congenial with the later strain (E. Tchougounova, G. Pejler and M. Abrink, The Journal of experimental medecine, vol. 198, no. 3, pp. 423-431, 2003) [ref. n°25]. The genotype of mMCP-4 KO mice used in the present study was confirmed by polymerase chain reaction (PCR) (as shown in supplementary Figure 1) via the use of primers described in Supplemental Table 1.
