# Supplementary material for: Significant Contribution of Mouse Mast Cell Protease 4 in Early Phases of Experimental Autoimmune Encephalomyelitis
Source: Mediators Inflamm. 2016 Aug 17;2016:9797021. doi: 10.1155/2016/9797021 (PMC5005578; doi:10.1155/2016/9797021)
Supplement: Supplementary file 1 — The mMCP-4 KO mice have been backcrossed for over 10 generations with C57BL/6 congeners and are highly congenial with the later strain (E. Tchougounova, G. Pejler and M. Abrink, The Journal of experimental medecine, vol. 198, no. 3, pp. 423-431, 2003) [ref. n°25]. The genotype of mMCP-4 KO mice used in the present study was confirmed by polymerase chain reaction (PCR) (as shown in supplementary Figure 1) via the use of primers described in Supplemental Table 1. [file 9797021.f1.docx]

**SIGNIFICANT CONTRIBUTION OF MOUSE MAST CELL PROTEASE 4 IN EARLY PHASES OF experimental AUTOIMMUNE ENCEPHALOMYELITIS**

Louisane Desbiens, Catherine Lapointe, Marjan Gharagozloo, Shaimaa Mahmoud, Gunnar Pejler, Denis Gris, and Pedro D’Orléans-Juste

**MEDIATORS OF INFLAMMATION**

**Supplementary Methods**

*DNA extraction and genotyping*

The end tip of the tail of WT and mMCP-4 KO mice was collected and the DNA was isolated and purified using the E.Z.N.A Tissue DNA Kit (Omega Bio-Tek Inc, Norcross, GA, USA) according to the manufacturer’s instructions. The genotype of the mice was then confirmed by PCR using increasing annealing temperatures in the following protocol: an initial denaturation for 2 min at 95°C, followed by 40 cycles of denaturation for 2 min at 95°C, annealing for 2 min 30 sec at 60 °C (+ 0.2 °C for each cycle), and extension for 6 min at 72°C, ending with a final 10 min at 72°C. The PCR contained 0.4 mM of dNTPs (Fermentas Canada Inc, Burlington, ON, Canada), 6 mM of MgCl_2_, 1 µM of each primer, 4 µl of the isolated DNA and 0.04 U/µl of recombinant Taq DNA polymerase (Life Technologies Inc, Grand Island, NY, USA) in Taq polymerase buffer 1X. The primers used are presented in Supplement Table 1. The final PCR products were migrated on a 2% agarose gel containing 200X SYBR Safe (Invitrogen, Carlsbad, CA, USA) for visualisation of the bands. WT alleles produce a 900 base pairs band while the KO alleles produce a fragment 380 bp.

**Supplementary Table 1 and Figure 1**

Supplementary Table 1 : Amplification conditions corresponding to the mMCP-4 gene for mouse genotyping used in this study.

| Products | Final Concentrations | Primers | | Amplification conditions | Temperature |
| --- | --- | --- | --- | --- | --- |
| Water  Buffer 10 X  dNTPs (10 mM)  MgCl_2_ (50 mM)  Primers (10 μM)  cADN  Taq pol. (1 U/μl) | --  1 X  0.4 mM/dNTP  6 mM  1 μM/primer  --  0.04 U/μl | 5’ – CAA GGT CCA ACT AAC TCC CTT TGT GCT CC – 3’ | Forward : common | Denaturation  Annealing  Éxtension | 95°C  60°C+0.2°C/cycle  72°C  (Nb cycle = 40) |
|  |  | 5’ – GGT GAT CTC CAG ATG GGC CAT GTA AGG GCG – 3’ | Reverse : WT gene |  |  |
|  |  | 5’ – GGG CCA GCT CAT TCC TCC CAC TCA TGA TCT – 3’ | Reverse : KO gene |  |  |

Amplified fragment length : 900 bp for WT gene and 380 bp for mutated gene.


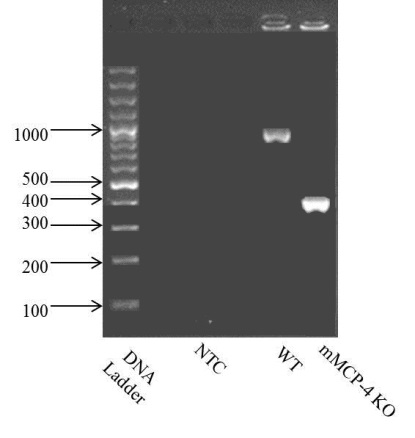


mMCP-4

KO

WT

NTC

DNA

Ladder

500

400

300

200

100

1000

Supplementary Figure 1: Genotyping of WT and mMCP-4 KO mice for the mMCP-4 gene. The bottom row indicates from which genotype the samples tested come from. The numbers at the left of the figure indicate the approximate size of double-stranded DNA on agarose gel. (NTC = no template control a negative control without DNA).
